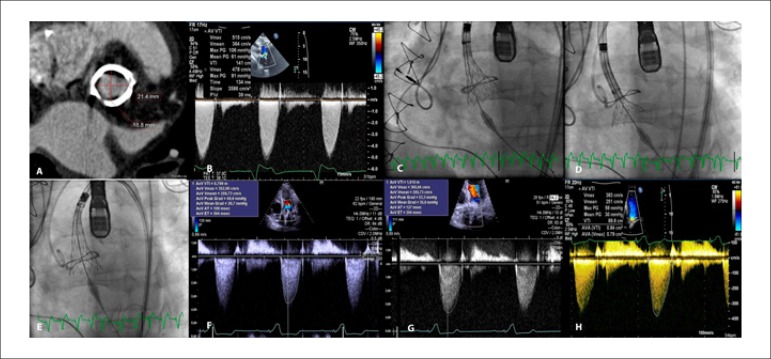# Transcatheter Valve-in-Valve Repositioning of CoreValve^®^ Evolut™ Rin Aortic Prosthesis

**DOI:** 10.5935/abc.20160007

**Published:** 2016-01

**Authors:** Ana Isabel Azevedo, Ricardo Ladeiras-Lopes, Alberto Rodrigues, Pedro Braga, Vasco Gama Ribeiro

**Affiliations:** Vila Nova de Gaia/Espinho Hospital Centre - Portugal

**Keywords:** Aortic Vave, Bioprosthesis, Heart Valve Prosthesis Implantation, Heart Failure

A 41-year-old man with history of surgical replacement of the aortic valve with a 21mm-Mitroflow bioprosthesis (1A), presented with functional class IV heart failure. Transesophageal echocardiography confirmed severe bioprosthesis obstruction (1B). We implanted a 23mm-CoreValve^®^ Evolut^™^ R (Medtronic, Minneapolis, USA) in the aortic bioprosthesis, by transfemoral approach. The valve was recaptured and repositioned during deployment (1C-E). Immediate (1F), one (1G) and three-month (1H) transthoracic echocardiography confirmed significant reduction in transaortic gradients. The patient remained in functional class I.

Our experience in repositioning the valve during a valve-in-valve procedure demonstrates the usefulness of this resource in such challenging procedures.

## Figures and Tables

**Figure:**